# Supplementary material for: A theory of cortical map formation in the visual brain
Source: Nat Commun. 2022 Apr 28;13:2303. doi: 10.1038/s41467-022-29433-y (PMC9050665; doi:10.1038/s41467-022-29433-y)
Supplement: Supplementary file 1 — Reporting Summary [file 41467_2022_29433_MOESM1_ESM.pdf]

Corresponding author(s): Jose Manuel Alonso

Last updated by author(s): Mar 8, 2022

# Reporting Summary

Nature Research wishes to improve the reproducibility of the work that we publish. This form provides structure for consistency and transparency in reporting. For further information on Nature Research policies, see our Editorial Policies and the Editorial Policy Checklist.

## Statistics

For all statistical analyses, confirm that the following items are present in the figure legend, table legend, main text, or Methods section.

| n/a | Confirmed | |
|---|---|---|
| ☐ | ☒ | The exact sample size (*n*) for each experimental group/condition, given as a discrete number and unit of measurement |
| ☐ | ☒ | A statement on whether measurements were taken from distinct samples or whether the same sample was measured repeatedly |
| ☐ | ☒ | The statistical test(s) used AND whether they are one- or two-sided<br>*Only common tests should be described solely by name; describe more complex techniques in the Methods section.* |
| ☐ | ☒ | A description of all covariates tested |
| ☒ | ☐ | A description of any assumptions or corrections, such as tests of normality and adjustment for multiple comparisons |
| ☐ | ☒ | A full description of the statistical parameters including central tendency (e.g. means) or other basic estimates (e.g. regression coefficient) AND variation (e.g. standard deviation) or associated estimates of uncertainty (e.g. confidence intervals) |
| ☐ | ☒ | For null hypothesis testing, the test statistic (e.g. *F*, *t*, *r*) with confidence intervals, effect sizes, degrees of freedom and *P* value noted<br>*Give P values as exact values whenever suitable.* |
| ☒ | ☐ | For Bayesian analysis, information on the choice of priors and Markov chain Monte Carlo settings |
| ☒ | ☐ | For hierarchical and complex designs, identification of the appropriate level for tests and full reporting of outcomes |
| ☐ | ☒ | Estimates of effect sizes (e.g. Cohen's *d*, Pearson's *r*), indicating how they were calculated |

*Our web collection on statistics for biologists contains articles on many of the points above.*

## Software and code

Policy information about availability of computer code

| | |
|---|---|
| Data collection | Data was collected with Omniplex Neural Data Acquisition Systems from Plexon while presenting stimuli generated with Matlab (versions: 2010 to 2014) and Psychtoolbox (versions: 3.0.9 to 3.0.11). All the electrophysiological measurements and computer simulations from this study are available from source data provided with this paper, from a repository in Zenodo , and upon request from the correspondence author (jalonso@sunyopt.edu). |
| Data analysis | All data analysis and computational modeling was performed using Matlab software (Matlab version 2020). The computational model was also tested with Matlab version 2021. Code to run customized simulations and generate the figures and tables reported in this study are available from a repository in Zenodo , and upon request from the correspondence author (jalonso@sunyopt.edu). |

For manuscripts utilizing custom algorithms or software that are central to the research but not yet described in published literature, software must be made available to editors and reviewers. We strongly encourage code deposition in a community repository (e.g. GitHub). See the Nature Research guidelines for submitting code & software for further information.

## Data

Policy information about availability of data

All manuscripts must include a data availability statement. This statement should provide the following information, where applicable:

- Accession codes, unique identifiers, or web links for publicly available datasets
- A list of figures that have associated raw data
- A description of any restrictions on data availability

All the electrophysiological measurements and computer simulations from this study are available from source data provided with this paper, from a repository in Zenodo , and upon request from the correspondence author (jalonso@sunyopt.edu).

# Field-specific reporting

Please select the one below that is the best fit for your research. If you are not sure, read the appropriate sections before making your selection.

☒ Life sciences ☐ Behavioural & social sciences ☐ Ecological, evolutionary & environmental sciences

For a reference copy of the document with all sections, see nature.com/documents/nr-reporting-summary-flat.pdf

# Life sciences study design

All studies must disclose on these points even when the disclosure is negative.

| | |
|---|---|
| Sample size | We performed the largest number of recordings possible to reach the statistical power needed to identify multiple significant relations among the different parameters of visual cortical maps. To test model predictions, the model generated sample sizes matching the data collected. |
| Data exclusions | To obtain accurate correlation values between ON-OFF receptive field dominance and orientation tuning, we selected receptive fields and visual responses with high signal to noise (RFsnr > 12, ORsnr > 6, ORresp > 10 spikes, see methods for further details and definitions of RFsnr, ORsnr, and ORresp). Visual responses and receptive fields that did not pass these pre-established criteria were excluded to avoid contaminating our measurements with noise. |
| Replication | The main findings were replicated in individual recording tracks within a cortical map, individual maps, and average across maps (in both data and model). The model always replicates the same cortical maps when the ONOFF retinal mosaics and afferent density are kept constant (Figure 3b). The experimental measures also replicate the correlations simulated by the model for the multiple stimulus dimensions represented in the cortical map (e.g. orientation tuning, clustering of orientation preference, spatial resolution, spatial frequency tuning). The replication with experimental measures is demonstrated by pooling together data collected from multiple multielectrode tracks and multiple animals (n= 8 animals, 17 single tracks, 633 data points, Figure 9e). It is also replicated in two individual tracks illustrated in Figure 9a-b. It is also replicated in measurements of correlation slopes illustrated in Figure 9g-h (n= 8 animals, 17 single tracks). |
| Randomization | We used all the data data collected to investigate the organization of visual cortical maps and test the computational model. In some figures of model simulations, we selected a random sample to allow visualization of individual data points (Figure 9c, 9f). The size of these random samples was adjusted to match the sample sizes collected in the experimental data. The stimulus presentation was randomized (see methods for details). |
| Blinding | The recordings that we performed in visual cortex and model simulations both require a detailed (non-blinded) investigation of all parameters involved in cortical map architecture. |

# Reporting for specific materials, systems and methods

We require information from authors about some types of materials, experimental systems and methods used in many studies. Here, indicate whether each material, system or method listed is relevant to your study. If you are not sure if a list item applies to your research, read the appropriate section before selecting a response.

## Materials & experimental systems

| n/a | Involved in the study | |
|---|---|---|
| ☒ | ☐ | Antibodies |
| ☒ | ☐ | Eukaryotic cell lines |
| ☒ | ☐ | Palaeontology and archaeology |
| ☐ | ☒ | Animals and other organisms |
| ☒ | ☐ | Human research participants |
| ☒ | ☐ | Clinical data |
| ☒ | ☐ | Dual use research of concern |

## Methods

| n/a | Involved in the study | |
|---|---|---|
| ☒ | ☐ | ChIP-seq |
| ☒ | ☐ | Flow cytometry |
| ☒ | ☐ | MRI-based neuroimaging |

## Animals and other organisms

Policy information about studies involving animals; ARRIVE guidelines recommended for reporting animal research

| | |
|---|---|
| Laboratory animals | Adult male cats, Felis catus (4-7 kg, 9 months to 1.5 years old) |
| Wild animals | No wild animals were used in the study |
| Field-collected samples | No field collected samples were used in the study |
| Ethics oversight | All surgical and experimental procedures were performed in accordance with the guidelines of the U.S. Department of Agriculture and were approved by the Institutional Animal Care and Use Committee at the State University of New York, State College of Optometry. |

Note that full information on the approval of the study protocol must also be provided in the manuscript.
