# Supplementary material for: A theory of cortical map formation in the visual brain
Source: Nat Commun. 2022 Apr 28;13:2303. doi: 10.1038/s41467-022-29433-y (PMC9050665; doi:10.1038/s41467-022-29433-y)
Supplement: Supplementary file 2 — Supplementary Information [file 41467_2022_29433_MOESM2_ESM.pdf]

A theory of cortical map formation in the visual brain
(Supplementary Figures and Tables)

Sohrab Najafian[1*], Erin Koch[1,2*], Kai Lun Teh[3-4], Jianzhong Jin[1], Hamed Rahimi-Nasrabadi[1], Qasim Zaidi[1], Jens Kremkow[3-4], Jose-Manuel Alonso[1]

[1]Department of Biological and Visual Sciences; SUNY College of Optometry, New York, NY, 10036, USA
[2]Division of Biology and Biological Engineering, Caltech, Pasadena, CA, 91125, USA.
[3]Neuroscience Research Center, Charité-Universitätsmedizin Berlin, Charitéplatz 1,10117 Berlin
[4]Bernstein Center for Computational Neuroscience Berlin, Philippstraße 13, 10115 Berlin

**Both authors contributed equally to this work*

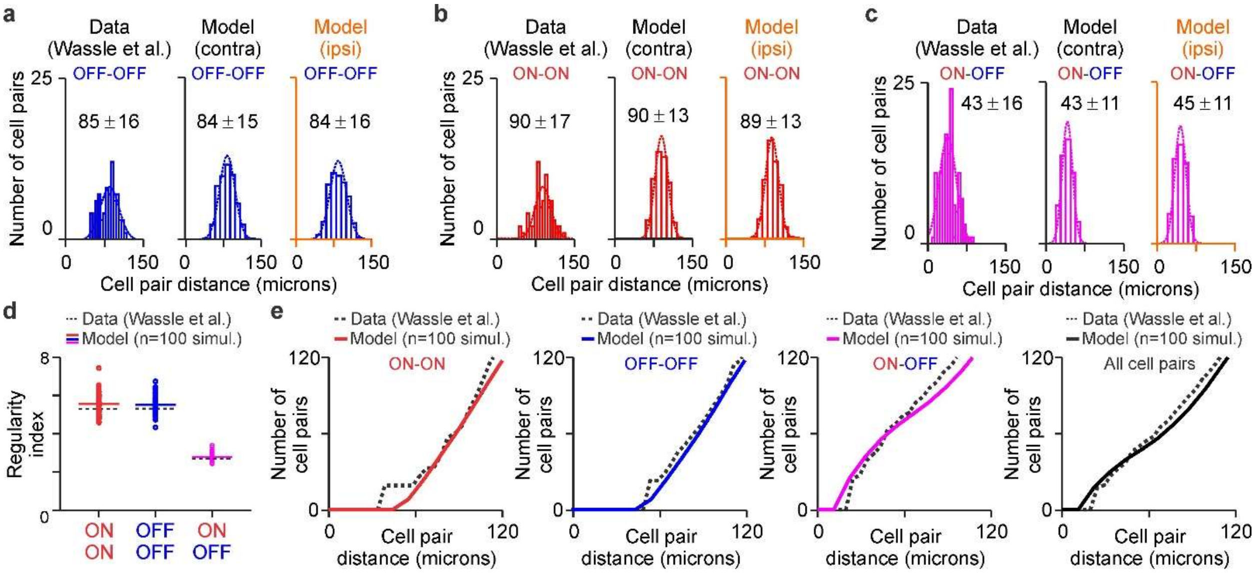

Supplementary Figure 1. Model simulations of cat retina. a. Distribution of retinal distance between pairs of the closest OFF beta retinal ganglion cells. Measurements from Wassle et al.[1] (left) and model simulations for the contralateral (middle) and ipsilateral eyes (right). **b.** Same as (a) for pairs of ON cells. **c.** Same as (a) for pairs of ON and OFF cells. **d.** Distribution and means of regularity indices for different cell pairs measured as the distribution mean divided by the standard deviation [2]. Measurements reported by Wassle et al.[1] are shown in dotted lines. Measurements of 100 model simulations are shown as small circles and their mean as horizontal lines for pairs of ON-ON cells (red), OFF-OFF cells (blue) and ON-OFF cells (purple). **e.** Distribution of cell pair distance for all possible pairs shown, from left to right, for ON-ON, OFF-OFF, ON-OFF, and all cell pairs, as in [2]. Data obtained from [1] by extracting the position of each retinal ganglion cell in the published retinal mosaic. Model measurements obtained from averages of 100 simulations. Source data are provided as a Source Data file.

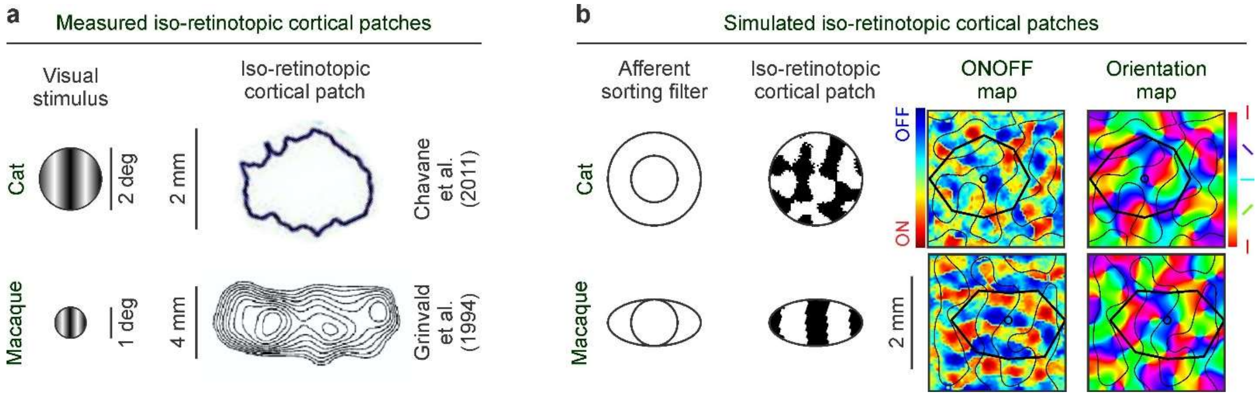

Supplementary Figure 2. Afferent sorting filters and iso-retinotopic cortical patches. a. Experimental measures of iso-retinotopic cortical patches measured with grating stimuli (left) in cats (right, top [3]) and macaques (right, bottom [4]). Notice that the iso-retinotopic cortical patch is more elongated in macaques than cats. **b.** Model simulations of iso-retinotopic cortical patches of cats (top) and macaques (bottom) obtained with different afferent sorting filters (left). The size and shape of the afferent shorting filter determines the size and shape of the iso-retinotopic cortical patch, which remains constant across the cortex and is superimposed with multiple ONOFF and orientation domains (right, see movies FigS2b_cat and FigS2b_macaque).

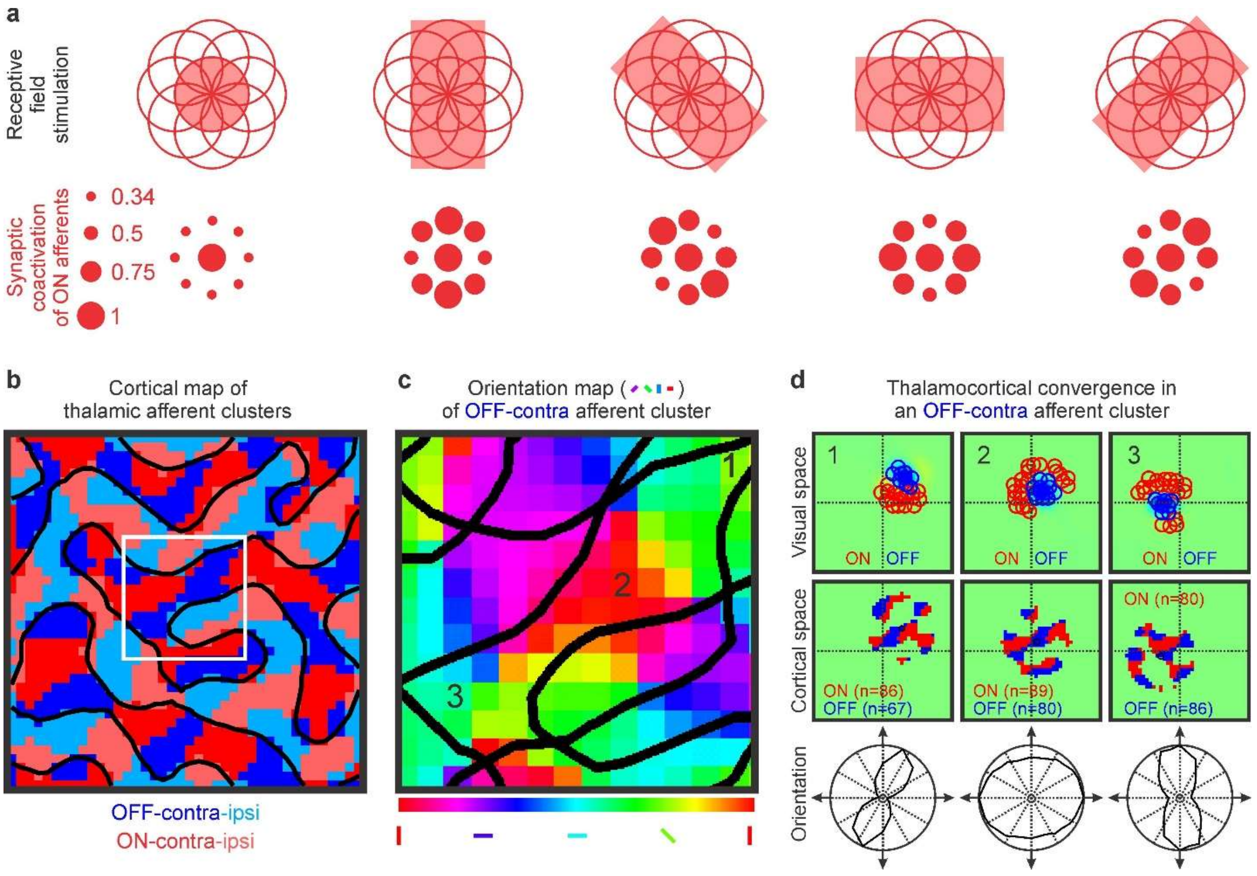

Supplementary Figure 3. Orientation coverage in afferent clusters. a. Visual stimuli with different orientations co-activate different sets of afferent receptive fields within each afferent cluster. In a geometrical array of nine circular receptive fields separated from each other by a circle radius, a centered visual stimulus only overlaps completely the central receptive field. Different stimulus orientations co-activate different sets of receptive fields (top) and the spatial overlap between the stimulus and each circular receptive field can range from 34 to 100% (bottom). **b-c.** Each afferent cluster (b) responds to multiple stimulus orientations (c). The orientation map in c shows the region of the cortical map marked with a white square in b. The black lines illustrate the borders between the afferent clusters shown in b. **d.** Thalamocortical convergence in visual space (top) and cortical space (middle) for three regions of the afferent cluster shown in b (1, 2, 3). The thalamocortical convergence in visual space (top) is illustrated by showing the receptive fields of the thalamic afferents converging at cortical pixel 1, 2 or 3 that had synaptic weights > 50% of maximum. The thalamocortical convergence in cortical space (middle) is illustrated by showing the cortical position of the afferent main axon trunks converging at cortical pixels 1, 2 or 3. The number of afferents is shown in parenthesis. The bottom of the figure illustrates the orientation tuning of each cortical pixel.

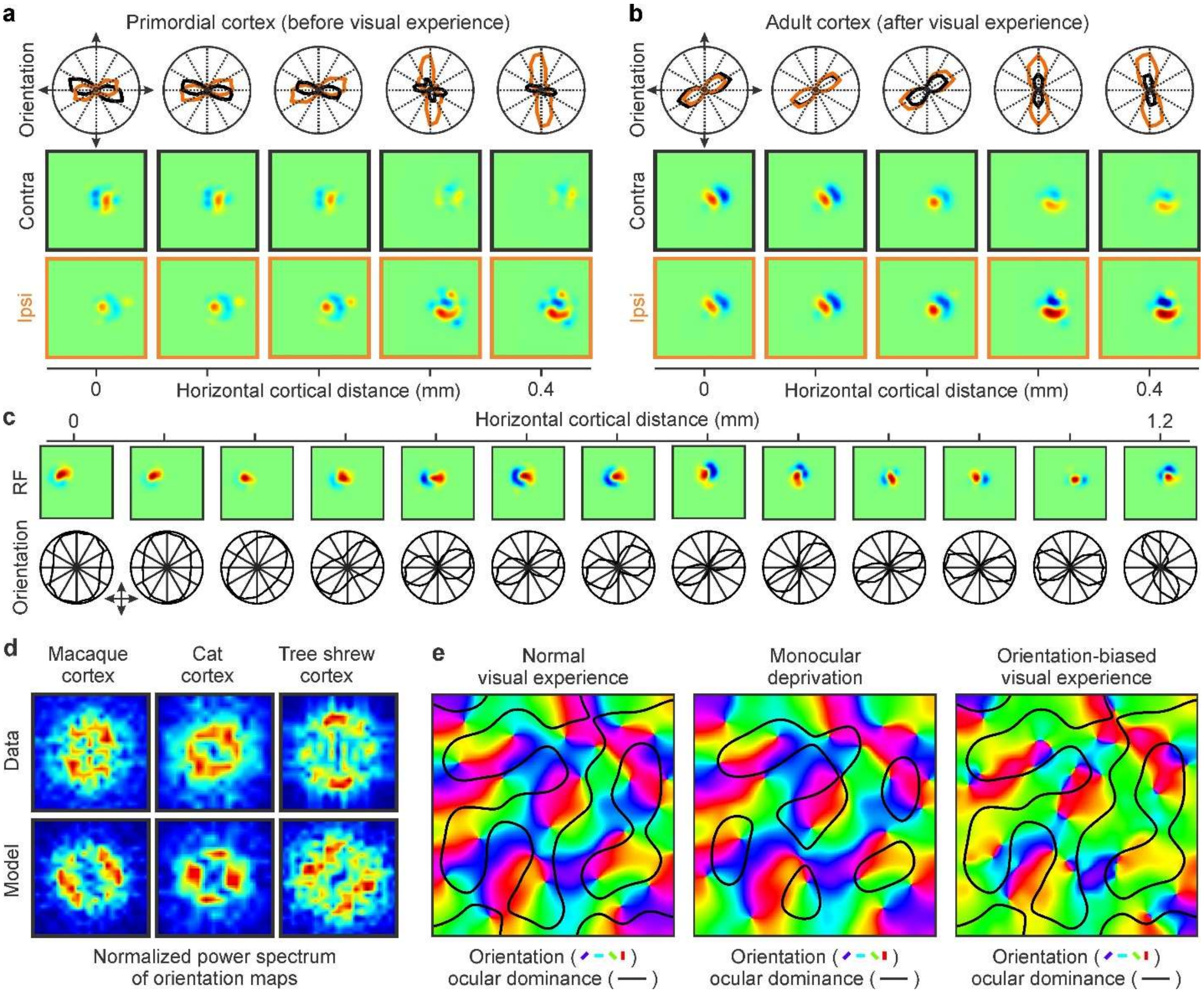

Supplementary Figure 4. Additional examples of model outputs. a. Model simulations of primordial visual cortex illustrating common binocular mismatches in orientation preference (top, contralateral eye in black, ipsilateral eye in orange) and receptive field structure (bottom square panels). **b.** Same as (a) but for mature visual cortex with accurate binocular matches in orientation preference and receptive field structure. **c.** Model simulations of changes in receptive field structure and orientation tuning with cortical distance. The orientation tuning is broadest at 0-0.2 mm and sharpest at 0.7-0.9. The model makes orientation preference to change slowly from 0 to 1.1 mm but can also reproduce rapid changes from 1.1. to 1.2 mm, described as 'fractures' in experimental measures[5]. **d.** Data-model comparisons of orientation map periodicity in normalized power spectrums. Data power spectrums were obtained from the images reproduced in Figure 7a. **e.** Model simulations of orientation map and ocular dominance borders (black lines) under normal visual experience (left), monocular deprivation (middle) and orientation biased visual experience (right).

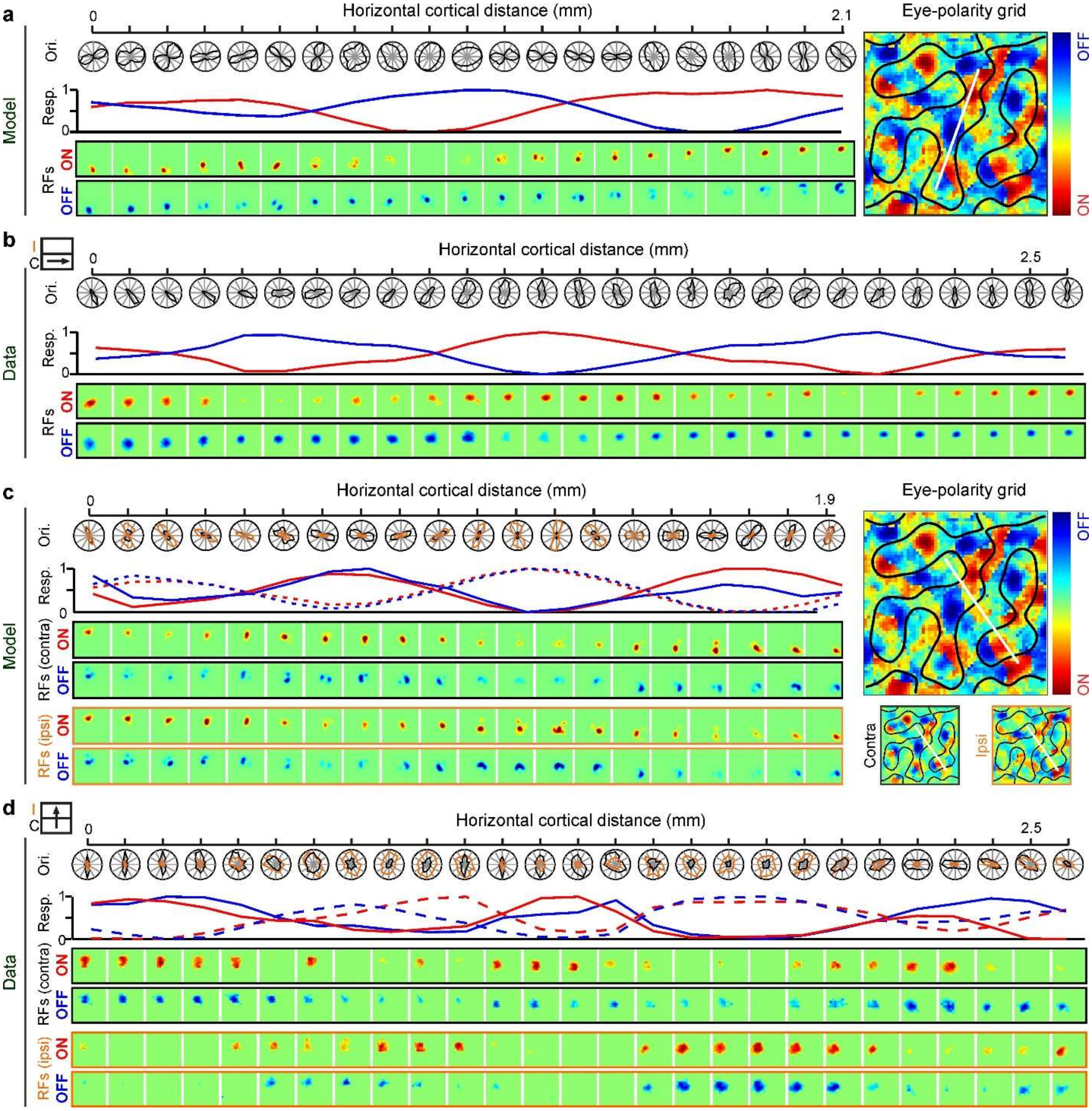

Supplementary Figure 5. Additional examples of model outputs. a. Model simulations of changes in orientation preference/tuning (top, left), ONOFF dominance (middle, left) and ONOFF retinotopy with horizontal cortical distance along a track running parallel to an ocular dominance band. The visual map on the right illustrates the track illustrated on the left (white line), the ON (red) and OFF (blue) cortical domains, and the border between ocular-dominance bands (black lines). **b.** Experimental measures of horizontal line tracks running parallel to an ocular dominance band in cat visual cortex. Reproduced from [6]. **c.** Same as (a) but for a track running perpendicular to ocular dominance bands. The right panels illustrate the tracks (white lines), ONOFF dominance (red-blue colors), and ocular dominance borders (black lines) in a simulated visual cortex mapped with binocular simulation (top), monocular stimulation of the contralateral eye (bottom left) and monocular stimulation of the ipsilateral eye (bottom right). **d.** Experimental measures of horizontal line tracks running orthogonal to an ocular dominance band in cat visual cortex. Reproduced from [6].

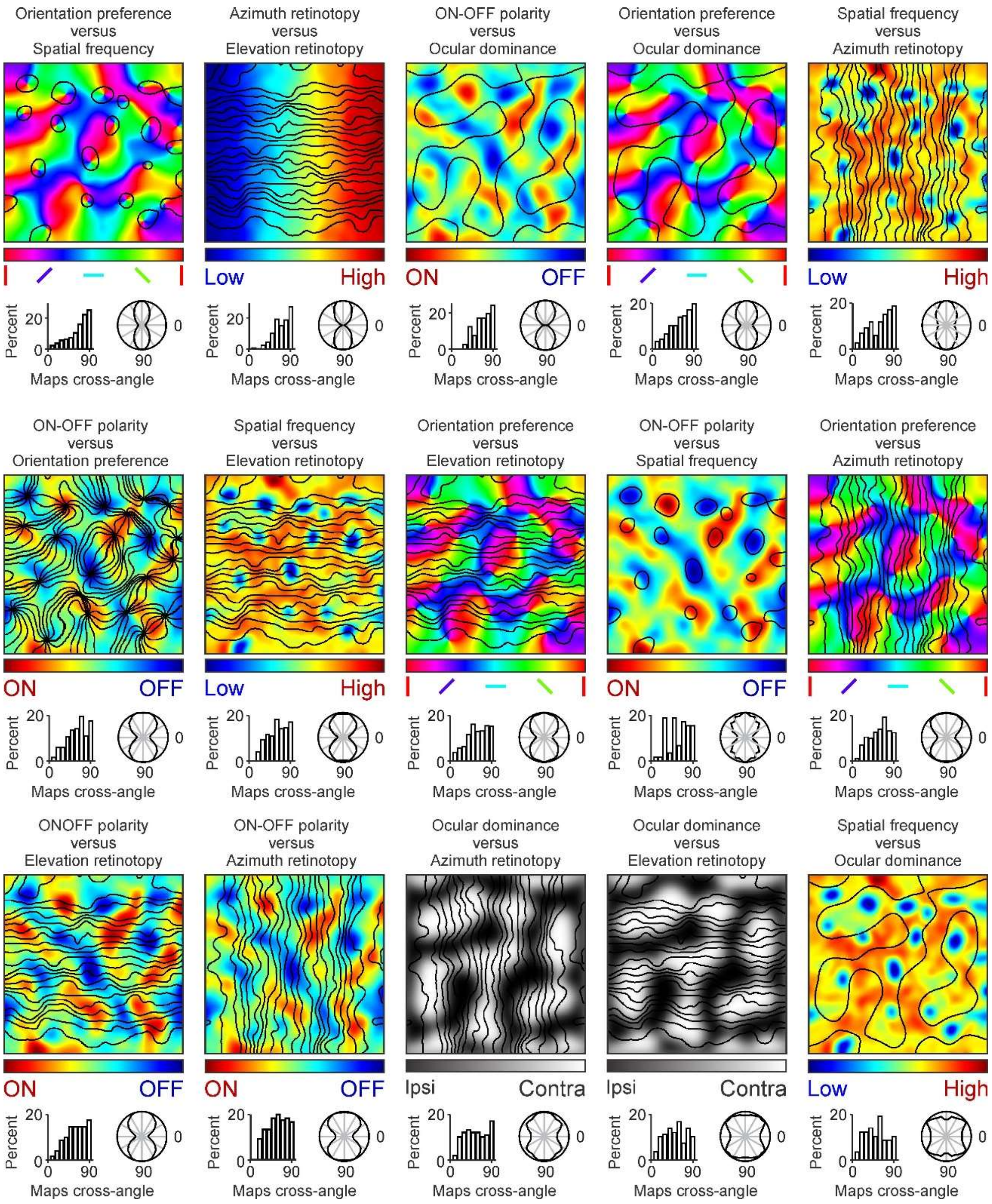

Supplementary Figure 6. Model simulations of relations between map gradients for different stimulus dimensions. For each map combination, the top panel shows pairs of superimposed maps (color scale for the map listed first and contour plot for the second). The bottom panels illustrate the intersection angles between iso-dimension lines of the two maps in a histogram (left) and a polar plot (right). From top left to bottom right, the map pairs are shown in order of 90-degree angle-dominance in the distribution of map intersection angles (top left: distribution with strongest 90-degree angle-dominance). Color scales show different orientations (orientation maps), retinotopy (azimuth and elevation maps), ON-OFF polarity (ON-OFF maps), eye input (ocular dominance maps), and spatial frequency (spatial-frequency maps). Contour plots show iso-orientation lines (orientation maps), iso-retinotopy lines (azimuth and elevation maps), iso-spatial-frequency lines (spatial frequency maps), and ocular dominance borders (ocular dominance maps). In spatial frequency maps, we use different contour levels for maps and histograms/polar-plots to better illustrate cortical regions with low spatial frequency and their angle crossings. In the maps, the iso-spatial frequency lines mark regions with a spatial frequency 35% lower than the maximum. In the histograms/polar-plots, the angle crossing includes both 26% and 44% iso-spatial-frequency lines.

	Map variable	Model error	Control error	p value
Macaque	Pinwheel density (pinwheels/mm ^{2})	2.55 ± 1.65	4.07 ± 0.96	0.56×10^{-10}
	Same-sign pinwheel distance (microns)	37.97 ± 33.69	222.5 ± 42.18	0.37×10^{-30}
	Opposite-sign pinwheel distance (microns)	43.46 ± 39.72	181.2 ± 50.99	0.47×10^{-28}
	Orientation domain peak cycle (microns)	58.85 ± 30.7	141.01 ± 46.03	0.65×10^{-14}
	Orientation domain valley cycle (microns)	172.99 ± 116.54	200.1 ± 67.725	0.45×10^{-10}
	Pattern similarity (1-r)	0.07 ± 0.01	0.13 ± 0.03	0.11×10^{-10}
	Gradient orthogonality	0.73 ± 1.78	1. 88 ± 0.76	0.14×10^{-5}
Cat	Pinwheel density (pinwheels/mm ^{2})	0.73 ± 0.48	4.07 ± 1.01	0.82×10^{-31}
	Same-sign pinwheel distance (microns)	85.75 ± 45.94	222.5 ± 37.99	0.51×10^{-29}
	Opposite-sign pinwheel distance (microns)	67.13 ± 50.62	181.2 ± 52.14	0.24×10^{-23}
	Orientation domain peak cycle (microns)	49.32 ± 38.76	138.62 ± 57.67	0.73×10^{-14}
	Orientation domain valley cycle (microns)	37.9 ± 49.05	194.56 ± 95.23	0.3×10^{-10}
	Pattern similarity (1-r)	0.11 ± 0.03	0.13 ± 0.02	0.52×10^{-3}
	Gradient orthogonality	0.38 ± 1.33	1.18 ± 0.8	0.14×10^{-4}
Tree shrew	Pinwheel density (pinwheels/mm ^{2})	1.83 ± 1.41	5.74 ± 1.57	0.69×10^{-26}
	Same-sign pinwheel distance (microns)	35.22 ± 29.85	282.1 ± 40.13	0.12×10^{-30}
	Opposite-sign pinwheel distance (microns)	40.91 ± 31.86	211.5 ± 56.47	0.28×10^{-29}
	Orientation domain peak cycle (microns)	22.81 ± 11.59	301.77 ± 52.137	0.33×10^{-15}
	Orientation domain valley cycle (microns)	228.36 ± 193.09	514.33 ± 104.84	0.52×10^{-13}
	Pattern similarity (1-r)	0.13 ± 0.03	0.27 ± 0.05	0.47×10^{-13}

Supplementary table 1. Quantification of model error at simulating same-species cortical maps. From left to right, species (first column), map properties (second column), and mean ± standard deviation of both model error (third column) and control error (fourth column). The fifth column shows the probability (p value) that the model error is lower than the control error (two-tailed Wilcoxon tests). Model errors were calculated as differences between simulated cortical maps and maps experimentally measured in animals from the same species. Control errors were calculated as differences between experimentally measured cortical maps from the two species with most similar brain sizes (macaque minus cat for macaque and cat map simulations, cat minus tree shrew for tree-shrew map simulations). Notice that the numbers in the fourth column for cat and macaque are very similar but not identical because the cat-macaque control errors were calculated twice, once for macaque and another for cat. Source data are provided as a Source Data file.

At least three correlations have values of r ≥						
	r ≥ 0.15	r ≥ 0.25	r ≥ 0.3	r ≥ 0.4	r ≥ 0.5	p value
Full model	996	989	974	929	827	$< 0.1 \times 10^{-300}$
No afferent sorting	727	524	179	25	2	
**All five correlations have values of r ≥**						
	r ≥ 0.15	r ≥ 0.25	r ≥ 0.3	r ≥ 0.4	r ≥ 0.5	p value
Full model	931	908	837	696	489	0.11×10^{-213}
No afferent sorting	122	47	6	0	0	
**All five correlations have correct sign and values of r ≥**						
	r ≥ 0.15	r ≥ 0.25	r ≥ 0.3	r ≥ 0.4	r ≥ 0.5	p value
Full model	929	908	837	696	489	0.11×10^{-213}
No afferent sorting	59	29	4	0	0	
**All five correlations have correct sign, range > 0.2, and values of r ≥**						
	r ≥ 0.15	r ≥ 0.2	r ≥ 0.3	r ≥ 0.4	r ≥ 0.5	p value
Full model	927	907	836	695	489	0.11×10^{-213}
No afferent sorting	7	3	0	0	0	

Supplementary table 2. Statistical significance of correlations for multiple stimulus dimensions. The table illustrates number of recording tracks out of 1000 simulated that had correlations meeting specific criteria. For each track, we measured the five correlations illustrated in figure 9 a-g among orientation selectivity, spatial resolution, low-pass index, and local orientation homogeneity. From left to right, the table illustrates simulation type (with or without afferent sorting for ocular dominance and ON-OFF polarity), number of correlations with specific r values, and statistical comparison between simulation types (p value obtained from counts for r ≥ 0.5 with a Chi squared test). From top to bottom, the table shows number of tracks with at least three correlations meeting the required strength, all five correlations meeting the required strength, all five correlations meeting the required strength and sign, and all the above criteria plus having a range of values within each correlation that is at least 0.2 times the range measured in experiments. Note that the number of tracks decreases as the criteria become stricter but the decrease is much more pronounced when the model does not sort afferents by eye input and ON-OFF polarity. Source data are provided as a Source Data file.

References for Supplementary information

1 Wassle, H., Boycott, B. B. & Illing, R. B. Morphology and mosaic of on- and off-beta cells in the cat retina and some functional considerations. *Proc R Soc Lond B Biol Sci* **212**, 177-195, doi:10.1098/rspb.1981.0033 (1981).

2 Eglen, S. J. & Wong, J. C. Spatial constraints underlying the retinal mosaics of two types of horizontal cells in cat and macaque. *Vis Neurosci* **25**, 209-214, doi:10.1017/S0952523808080176 (2008).

3 Chavane, F. *et al.* Lateral Spread of Orientation Selectivity in V1 is Controlled by Intracortical Cooperativity. *Front Syst Neurosci* **5**, 4, doi:10.3389/fnsys.2011.00004 (2011).

4 Grinvald, A., Lieke, E. E., Frostig, R. D. & Hildesheim, R. Cortical point-spread function and long-range lateral interactions revealed by real-time optical imaging of macaque monkey primary visual cortex. *J Neurosci* **14**, 2545-2568 (1994).

5 Blasdel, G. G. Orientation selectivity, preference, and continuity in monkey striate cortex. *J Neurosci* **12**, 3139-3161 (1992).

6 Kremkow, J., Jin, J., Wang, Y. & Alonso, J. M. Principles underlying sensory map topography in primary visual cortex. *Nature* **533**, 52-57, doi:10.1038/nature17936 (2016).
